# Quantitative Structure Activity Relationship of Cinnamaldehyde Compounds against Wood-Decaying Fungi

**DOI:** 10.3390/molecules21111563

**Published:** 2016-11-17

**Authors:** Dongmei Yang, Hui Wang, Haijian Yuan, Shujun Li

**Affiliations:** Key Laboratory of Bio-Based Material Science and Technology of the Ministry of Education, Northeast Forestry University, Harbin 150040, China; 13654659109@163.com (D.Y.); wh-snowis@outlook.com (H.W.); yhj_20077@163.com (H.Y.)

**Keywords:** cinnamaldehyde, derivatives, QSAR models, design, wood-decaying fungi

## Abstract

Cinnamaldehyde, of the genius *Cinnamomum*, is a major constituent of the bark of the cinnamon tree and possesses broad-spectrum antimicrobial activity. In this study, we used best multiple linear regression (BMLR) to develop quantitative structure activity relationship (QSAR) models for cinnamaldehyde derivatives against wood-decaying fungi *Trametes versicolor* and *Gloeophyllun trabeum*. Based on the two optimal QSAR models, we then designed and synthesized two novel cinnamaldehyde compounds. The QSAR models exhibited good correlation coefficients: *R*^2^*_Tv_* = 0.910 for *Trametes versicolor* and *R*^2^*_Gt_* = 0.926 for *Gloeophyllun trabeum*. Small errors between the experimental and calculated values of two designed compounds indicated that these two QSAR models have strong predictability and stability.

## 1. Introduction

Wood, an extremely common and multi-purpose material, is susceptible to corrosion and degradation by fungal rot [1]. For practical application, wood is typically processed with preservatives to lengthen its life cycle. Traditional wood preservatives are Copper Chrome Arsenic (CCA), Copper Chrome Boron (CCB), Ammoniacal Copper Quate (ACQ), etc. Most of them consist of copper, chromium or arsenic compounds and their metal salts, which have a serious impact on human health and the environment. Consequently, most European countries have strictly limited the use of chromium and arsenic-based wood preservatives, especially in children’s playground equipment and garden furniture [2,3]. Natural wood preservatives as an alternative have attracted a great deal of research [4]. Some specific woods or plants have the ability to self-protect to resist decay caused by fungi and insects, like cinnamon. The effective material in cinnamon is cinnamaldehyde, which is extracted from the bark and leaves of cinnamon trees [5,6].

Cinnamaldehyde exhibits extensive antimicrobial abilities, particularly in regards to inhibiting the growth of fungi and gram-positive bacterium [7,8]. The aforementioned antimicrobial capability is largely due to an aldehyde group conjugated with a benzene ring in cinnamaldehyde’s structure [9,10]. This aldehyde group is a nucleophilic group that is easily absorbed by the hydrophilic group on the surfaces of bacteria and, once across the cell wall, begins a process of inhibition and sterilization by destroying the bacteria’s polysaccharide structure. Because mammalian cells lack cell walls, cinnamaldehyde is safe for humans and their environment when used as a wood preservative [7,9].

There are negative consequences associated with the use of cinnamaldehyde as a wood preservative, however. First, its poor water solubility can cause few kinds solvents to permeate into the wood material [11]; second, it has high volatility and a strong smell, which limits its long-term application [12]. In this study, we endeavored to add to the limited research concerning cinnamaldehyde derivatives by exploring the relationship between their structure and antifungal activity against *Trametes versicolor* and *Gloeophyllun trabeum*. Then, QSAR models were established and those models provided a basic theoretical frame work for the application of cinnamaldehyde derivatives as a wood preservative. According to the QSAR models, two new cinnamaldehyde derivatives with satisfactory antifungal activity against two wood-decaying fungi were designed and tested, which could be used to validate the predictability of the QSAR models.

## 2. Results and Discussion

### 2.1. Determining Optimal QSAR Models against Trametes versicolor and Gloeophyllun trabeum

#### 2.1.1. Establishing Optimal QSAR Models

**Figure 1:**
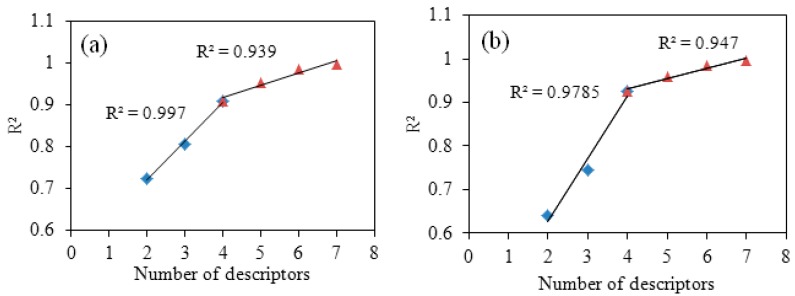
Breaking point rule for determining the number of descriptors ((**a**) is breaking point rule for *Trametes versicolor*; (**b**) is breaking point rule for *Gloeophyllun trabeum*).

“Breaking point” method was used to determine the optimal QSAR models of cinnamaldehyde compounds against *Trametes versicolor* and *Gloeophyllun trabeum* as shown in Figure 1. The *x*-coordinate represents number of descriptors, and the *y*-coordinate represents the correlation coefficient *R*^2^ of the corresponding model. As the trend line shows: the correlation coefficient *R*^2^ increased as the number of descriptors increased. When the number of descriptors (*n*) was less than 4, the correlation coefficient *R^2^* increased sharply. The fitting line with high correlation coefficient is 0.997 and 0.9785. When the number of descriptors exceeded 4, the correlation coefficient *R*^2^ increased slightly. The fitting line also had a high correlation coefficient 0.939 and 0.947. According to this method, the breaking point appeared when the number of descriptors was 4 or higher, as shown in Figure 1. The number of descriptors of the best models should also meet the requirements of multi-linear regression, as evidenced by the number of descriptors (*k*) of the optimal models and the sample number (*n*) ≥ 3(*k* + 1) [13]. Therefore, the number of descriptors of the optimal QSAR models against *Trametes versicolor and Gloeophyllun trabeum* is 4. The value of optimal descriptors is shown in Table 1 and Table 2.

The optimal models are shown in Table 3 and Table 4, these models had the following statistical characteristics: *R*^2^ = 0.910, *F* = 35.32, and *s*^2^ = 0.0093 for *Trametes versicolor*; *R*^2^ = 0.926, *F* = 43.95, and *s*^2^ = 0.0049 for *Gloeophyllun trabeum*.

**Figure 2:**
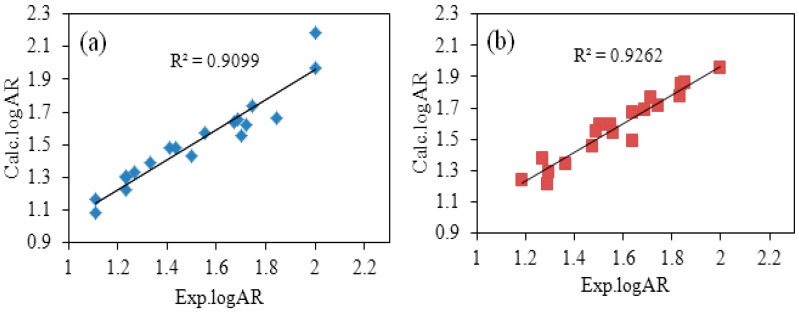
Experimental logAR versus predicted logAR according to the best QSAQ model ((**a**) is for *Trametes versicolor*; (**b**) is for *Gloeophyllun trabeum*).

Table 5 shows a comparison between experimental values (Exp.logAR) and calculated values (Calc.logAR). And the plot of Exp.logAR versus Calc.logAR is shown in Figure 2. The Calc. logAR was calculated according to the optimal QSAR models. There was little difference among Calc.logAR and Exp.logAR, demonstrating that calculated values were close to the experimental values at averages of 0.0661 and 0.0465, respectively, as shown in Table 5. This miniscule difference indicated that the optimal QSAR models are capable of accurately describing the relationship between chemical structure and bioactivity.

#### 2.1.2. Validation of Optimal QSAR Models

The internal validation results of the optimal QSAR models against *Trametes versicolor* and *Gloeophyllun trabeum* are shown in Table 6. The training set models for *Gloeophyllun trabeum* had the following characteristics: *R*^2^(fit) ≥ 0.900, *F*(fit) ≥ 18.03, *s*^2^(fit) ≤ 0.0057 for *Gloeophyllun trabeum; R*^2^(fit) ≥ 0.909, *F*(fit) ≥ 20.07, *s*^2^(fit) ≤ 0.0078 for *Trametes versicolor*, and the average correlation coefficient were 0.932 and 0.929, respectively. Each test set compound was predicted according to the above training test models, then compared and evaluated according to the predicted and experimental values by linear fitting. The results for linear fitting showed that the average correlation coefficient (*R*^2^ (pred)) was 0.833 and 0.792, respectively. All the internal validation results indicated that the optimal QSAR models are predictable and stable in effect.

As described in Section 3.2.2, the optimal QSAR models were subjected to external validation; the correlation coefficient of the external validated models were *R*^2^*_Tv_* = 0.948 and *R*^2^*_Gt_* = 0.926. The last compounds were predicted by the above external validated models. In the linear fitting of the predicted and experimental values of last compounds, the correlation coefficients were 0.804 and 0.984, respectively. These results also demonstrated that the optimal QSAR models had good predictability [14].

According to the external and internal validation tests, the optimal QSAR models were those which could be described using mathematical equations. The optimal QSAR models of the cinnamaldehyde derivatives against *Trametes versicolor* and *Gloeophyllun trabeum* were best described using Equations (1) and (2).

logAR_Tv_ = (0.2608 ± 0.4278) − (5.8562 ± 0.6297) × d1 − (28.275 ± 3.3560) × d2 − (0.0912 ± 0.0196) × d3 + (2.5481 ± 0.6368) × d4 (1)

logAR_Gt_ = (1.5166 ± 0.0591) − (5.8328 ± 0.508) × d1 − (0.1190 ± 0.0170) × d2 − (8.0388 ± 1.3311) × d3 − (11.201 ± 2.8386) × d4 (2)

#### 2.1.3. Descriptor Analysis in the Optimal QSAR Models

A *t*-test is typically utilized to measure the importance of descriptors in correlation [15]. According to the *t*-test values in Table 3, the most statistically significant descriptor is the minimum net atomic charge for an H atom, d1. This is a quantum chemical descriptor that indicates the hydrogen-bond and electrostatic interaction between negative ion and positive ion [16]. In Table 3, the positive correlation coefficient for d1 demonstrated that increasing the hydrogen-bonding and electrostatic interaction in cinnamaldehyde derivatives led to an increase in antifungal activity against *Trametes versicolor* [17].

The second descriptor was FNSA-3 fractional PNSA (PNSA-3/TMSA), d2 [18], which is the ratio of PNSA-3 and TMSA that can be computed as follows [19]:

(3) FNSA−3=PNSA−3/TMSA

where TMSA is total area of the molecule and PNSA-3 is the atomic charge weight of the negatively charged molecular surface area [20]:

(4) $$\mathrm{PNSA}-3=\sum \text{​}q_{A}S_{A}\;\;\;\;\;A\in \left\{\delta_{A}<0\right\}$$

where q_A_ is the partial charge of the atom and S_A_ is the respective atomic negatively charged solvent-accessible surface area. Both q_A_ and S_A_ were computed in Codessa. FNSA-3 is a significant factor on polar active and hydrogen-bond active charges.

The third descriptor was ESP-RPCS relative charged SA (SAMPOS*RPCG) (Quantum-Chemical PC), d3, which is also a quantum chemical descriptor. This descriptor reflects the total molecular surface area and properties of the function group and indicates interactions among polar molecules [18].

The fourth descriptor was YZ Shadow/YZ Rectangle, d4, a space property descriptor [21]. The YZ Shadow was calculated by projecting a molecule on the YZ plane, which is related to molecular conformation and molecular orientation. This shape parameter provided a positive indication of the antifungal activity of the cinnamaldehyde derivatives. As the value of descriptor YZ Shadow increased, the antifungal activity of cinnamaldehyde derivatives against *Trametes versicolor* also increased.

As shown in Table 4, the most statistically significant descriptor was the ESP minimum net atomic charge for an H atom, d1 for QSAR model against *Gloeophyllun trabeum*. The second most important descriptor was ESP-RPCS Relative positive charged SA(SAMPOS*RPCG) (Quantum-Chemical PC), d5, which is similar to ESP-RPCS Relative charged SA (SAMPOS*RPCG) (Quantum-Chemical PC), d3. It is the result of the partial positive charged surface multiplied by the relative positive charge [18]. The third and fourth most important descriptors were FNSA-3 (PNSA-3/TMSA) (Quantum-Chemical PC), d6, and FNSA-3Fractional PNSA (PNSA-3/TMSA), d7. These are quantum chemical descriptors which describe the total molecular surface properties and the functional group as well as the activity of polar molecules [19].

#### 2.1.4. Designing the New Compound with High Bioactivity, Calculating Its AR

**Figure 3:**
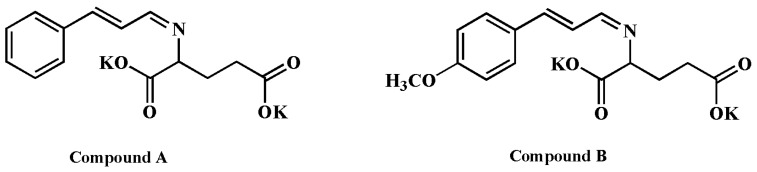
Structures of designed compounds.

Two cinnamaldehyde amino acid Schiff base compounds with satisfactory predicted activities were selected to synthesize and test their antifungal activity, the structures of those two designed compounds were shown in Figure 3. The chemical structures of new compounds were confirmed by ^1^H-NMR, IR, MS, HPLC, purity and melting point. The antifungal activity of new compounds was tested by the same method described in Section 3.2.1, and antifungal activity ratio (**AR**) of two designed compounds were listed in Table 7.

*Cinnamaldehyde glutamic acid potassium Schiff base* (Compound **A**). Orange powder; m.p. 233.7–236.5 °C; purity: 91.2817%; IR (cm^−1^): 1631 (C=O), 1588 (C=N, C_arom_=C_arom_), 1492 (C_arom_=C_arom_), 754 (Ar-H), 689 (Ar-H); ^1^H-NMR (400 MHz, D_2_O) δ 7.83 (t, *J* = 10.0 Hz, 1H, CH=N-), 7.35 (dd, *J* = 12.3, 10.8 Hz, 2H, Ar-H), 7.19–7.10 (m, 3H, Ar-H), 6.89 (d, *J* = 16.0 Hz, 1H, CH=C-), 6.82–6.71 (m, 1H, C=CH-), 3.53 (dd, *J* = 8.5, 4.9 Hz, 1H, -CH-), 2.10–2.00 (m, 1H, -CH–C), 2.00–1.90 (m, 2H, -CH_2_-COOK), 1.89–1.80 (m, 1H, -CH-); MS *m*/*z* calcd. for C_14_H_13_K_2_NO_4_ 337.0. [M + H]^+^, found [M + K]^+^ 376.2.

*P-Methoxy cinnamaldehyde glutamic acid potassium Schiff base* (Compound **B**). Orange powder; m.p. 241.4–244.5 °C; purity: 83.749%; IR (cm^−1^): 1633 (C=O), 1589 (C=N, C_arom_=C_arom_), 1520 (C_arom_=C_arom_), 816 (Ar-H); ^1^H-NMR (400 MHz, D_2_O) δ 8.02 (d, *J* = 9.0 Hz, 1H, CH=N-), 7.52–7.46 (m, 2H, Ar-H), 7.04 (d, *J* = 15.9 Hz, 1H, Ar-H), 6.95–6.90 (m, 2H, CH=C-), 6.83 (dd, *J* = 15.9, 9.0 Hz, 1H, C=CH-), 3.81 (s, 3H, Ar-OCH_3_), 3.71 (dd, *J* = 8.6, 5.0 Hz, 1H, -CH-COOK), 2.31–2.24 (m, 1H, -CH-), 2.19–2.10 (m, 2H, COOK-CH_2_-), 2.04 (d, *J* = 13.0 Hz, 1H, -CH-); MS *m*/*z* calcd. for C_15_H_15_K_2_NO_5_ 367.0. [M + H]^+^, found [M + K]^+^ 406.3.

**Figure 4:**
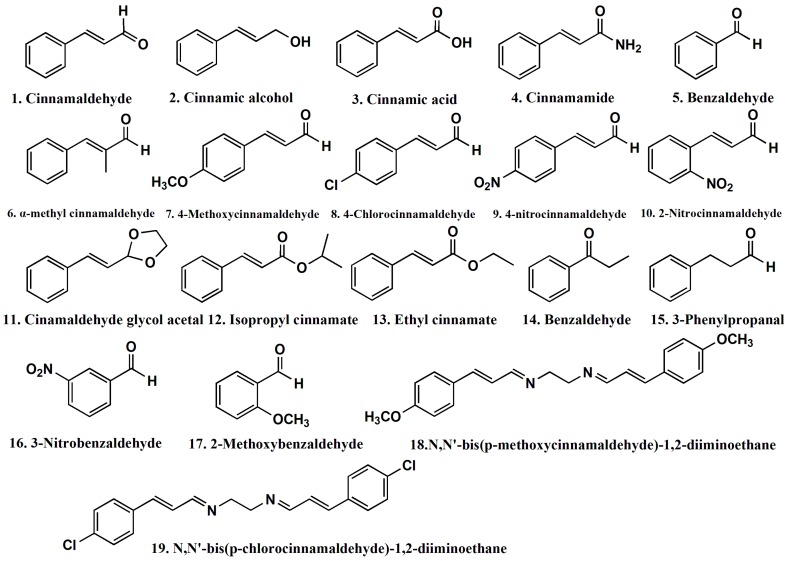
Structures of 19 cinnamaldehyde derivatives.

As shown in Table 7, the designed compounds exhibited better antifungal qualities than the 19 cinnamaldehyde compounds listed in Figure 4. The *AR_Gt_* of the new compounds against *Gloeophyllun trabeum* exceeded the *AR_Tv_* against *Trametes versicolor*, indicated that the new compounds possessed better antifungal properties than cinnamaldehyde alone. Additionally, the antifungal activity of the new compounds against *Gloeophyllun trabeum* significantly exceeded the AR of cinnamaldehyde alone. Concerning the experimental logAR and calculated logAR from optimized models, the experimental value was close to the calculated value for both compounds against both fungi. The smallest error was 0.0155 for Compound A against *Gloeophyllun trabeum*. This suggested that the QSAR model against *Gloeophyllun trabeum* exhibited stronger predictability and stability, with a higher correlation coefficient (*R*^2^ = 0.926) and better validation results than models against *Trametes versicolor* (*R*^2^ = 0.910).

## 3. Materials and Methods

### 3.1. Materials

Analytical-grade reagents included ethanol, acetone, cinnamic acid, benzaldehyde, and acetophenone. Cinnamon oil (cinnamaldehyde content, 95%) was produced by the Zhenxing spices oil refinery of Ji’an City, Jiangxi Province, China. Industrial-grade reagents included cinnamamide, 2-methyl-3-phenylacrylaldehyde, 3-phenylpropanal, 3-(4-Chlorophenyl) acrylaldehyde, 4-methoxycinnamaldehyde, 3-(4-nitrophenyl)acrylic acid, 3-(2-Nitrophenyl)acrylaldehyde, cinnamaldehyde glycol acetal, 2-methoxybenzaldehyde, 3-nitrobenzaldehyde, isopropyl cinnamate, and ethyl cinnamate and were produced by Wuhan Yuancheng Technology Development, Wuhan, China. *N*,*N*′-bis (*p*-methoxycinnamaldehyde)-1,2-diiminoethane and *N*,*N*′-bis (*p*-chlorocinnamaldehyde)-1,2-diiminoethane were synthesized in the laboratory per the instructions in references [22]; the purity of these compounds exceeded 95%. Sodium chloride and glucose were produced by Tianjin Damao Chemical Reagent Factory, Tianjin, China. Peptone and beef extract were produced by Beijing Aoboxing Biotechnology LLC (Beijing, China). Instrumentation included an electro-heating standing-temperature cultivator (DHP-9162), sterilizer (YX280A), and bench top (SW-CJ-2FD).

### 3.2. Method

#### 3.2.1. Paper Disc Method

The paper disc method was used to determine the antifungal activity for cinnamaldehyde compounds [23]. Two wood-decaying fungi, *Trametes versicolor* and *Gloeophyllun trabeum*, were used as the test microorganisms after cultivation for two days at 30 °C [24]. The concentration of cinnamaldehyde compounds used in the experiment was 0.25 mol/L.

The medium, paper disc with diameter 8 mm, 0.9 wt % normal saline and petri dishes were sterilized 30~35 min under high pressure and temperature. All the vessels and instruments were subjected to ultraviolet germicidal irradiation for 20 min. Then, 10 mL of the melted medium was transferred into each petri dish and allowed to solidify. After that, 125 μL microorganism suspension was spread on solid medium. And the paper disc impregnated with 0.25 mol/L cinnamaldehyde derivatives solution, were placed in the center of the petri dishes. At last, the petri dishes were cultivated in a constant temperature cultivator (incubator) at 30 °C for 2–3 days. The antifungal activity was determined by measuring the inhibition zones around the discs, the larger the inhibition zone, the greater antifungal activity. All tests were performed in triplicate. 

The cinnamaldehyde served as the control. The antifungal activity ratio of cinnamaldehyde derivatives were described using the following equation [8,24]:

(5) AR=(dd0)×100%

where *d* is the average inhibition zone of the cinnamaldehyde derivatives, and *d*_0_ is the average inhibition zone of cinnamaldehyde. The antifungal activity rates and their two-dimensional structure of the 19 cinnamaldehyde derivatives are shown in Figure 4.

#### 3.2.2. Establishing QSAR Models

There were three steps for establishing the QSAR models of the cinnamaldehyde derivatives [12].

(1) Molecule structure geometry optimization: By ChemDraw3D software, the structures of 19 cinnamaldehyde compounds were drawn, and their three-dimensional structures were initially optimized geometrically using the MM^2+^ function. The initial optimized structures were inputted in AMPAC Agui 9.2.1 software to conduct geometric optimizing.
(2) Descriptor calculation: In Codessa 2.7.16 software, 4 kinds of descriptors could be calculated for a molecular, Molecule descriptor, Fragment descriptor, Pair and Atom descriptor. In this paper, optimal structures of cinnamaldehyde derivatives were inputted into Codessa 2.7.16 software to calculate Molecule descriptors. These descriptors were divided into six groups: structural, topological, geometrical, thermodynamic, electrostatic, and quantum-chemical descriptors. All were involved in this paper with the exception of thermodynamic descriptor. These descriptors were the basis for establishing the QSAR models [25].
(3) The establishing for best QSAR model: The Best Multi-Linear Regression equation was built by Codessa 2.7.16 software [26]. After Best Multi-Linear Regression analysis, a series of QSAR models were developed. A general method “breaking point” was used to determine the number of descriptors by searching the breaking point of the two *R*^2^ trend lines. The relationship between *R^2^* and number of descriptor were described as Figure 1 [27]. Two different solutions were used to validate the best models and to explore predictability and stability–internal validation and external validation, respectively.

#### 3.2.3. Validating QSAR Models

Internal validating: The 19 compounds were divided into three groups A (1, 4, 7, 10…), B (2, 5, 8…), and C (3, 6, 9…). Each coupled groups (A + B, B + C, and A + C) was combined as the training set, and the individual group as the test set (C, A, and B). The training set was inputted to Codessa software to develop new four-descriptor QSAR model, then used these models to predict the bioactivity of the group (test set) that had been left out. This was done for each coupled group (A + B, B + C, and A + C). The predicted AR and experimental AR of each testing set compounds were linear fitted by Origin Pro 8.0 software with fixed slope. A series of results *R*^2^, *s*^2^, and *F* values of each training set and testing set were listed in Table 6 [28].

External validation was determined using a similar validation method [29]. Four of 19 compounds were chosen as the external set, and the other compounds as the training set. Training set compounds were inputted to Codessa to establish four-descriptor QSAR models, then QSAR models were used to predict the external set.

#### 3.2.4. Design of New Compounds

**Figure 5:**
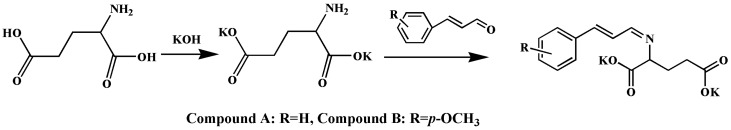
Synthesis routes of novel compounds.

Cinnamaldehyde amino acid Schiff base compounds are novel compounds with good water solubility, very weak odor, and good bioactivity [10,30]. Several kinds of cinnamaldehyde amino acid Schiff base compounds were designed. The structures of designed compounds were drawn by ChemDraw 3D software and optimized by AMPAC Agui 9.2.1 software. Then the optimal geometric molecular structures of designed compounds were inputted to Codessa to calculate the molecule descriptor and predict logAR by the best QSAR models. The logAR values of the designed compounds were screened, and two designed compounds had higher logAR value than cinnamaldehyde. Finally, two designed compounds A and B were synthesized as Figure 5 shows [30]. The AR of the two designed compounds was determined as described in Section 3.2.1.

## 4. Conclusions

In this study, two optimal QSAR models of cinnamaldehyde derivatives against wood-decaying fungi were established and validated, with the following statistical characteristics: *R*^2^ = 0.910, *F* = 35.32, and *s*^2^ = 0.0093 for *Trametes versicolor*; *R*^2^ = 0.926, *F* = 43.95, and *s*^2^ = 0.0049 for *Gloeophyllun trabeum*. There were seven main parameters effecting antifungal activity of cinnamaldehyde compounds in QSAR models: ESP minimum net atomic charge for an H atom, FNSA-3 Fractional PNSA (PNSA-3/TMSA), ESP-RPCS Relative charged SA (SAMPOS*RPCG), YZ Shadow/YZ Rectangle, ESP-RPCS Relative positive charged SA (SAMPOS*RPCG), FNSA-3 (PNSA-3/TMSA), and FNSA-3 Fractional PNSA (PNSA-3/TMSA). Two new cinnamaldehyde amino acid compounds were designed and synthesized on the basis of these QSAR models and obtained satisfactory results, as the experimental logAR was extremely close to the calculated logAR. The errors were smaller (and thus the model more predictable) for *Gloeophyllun trabeum* than the errors for *Trametes versicolor*, but taken together, internal and external validation results reflect a level of predictability in our QSAR models that is highly consistent. In summary, this study showed that QSAR models of cinnamaldehyde derivatives can be used to predict the antifungal activity of new cinnamaldehyde compounds against wood-decaying fungi.

## Figures and Tables

**Table 1:** Antifungal activity ratios (AR) and descriptors of cinnamaldehyde analogues for *Trametes versicolor*.

ID	AR	logAR	ESP-Min Net Atomic Charge for a H Atom, d1	FNSA-3 Fractional PNSA (PNSA-3/TMSA), d2	ESP-RPCS Relative Charged SA (SAMPOS*RPCG), d3	YZ Shadow/YZ Rectangle, d4
**1**	100	2	0.0585	−0.0241	1.3522	0.7903
**2**	25.71	1.4102	0.0966	−0.0201	0.8218	0.7114
**3**	31.43	1.4973	0.1701	−0.0271	0.7785	0.7839
**4**	17.14	1.2341	0.1632	−0.0213	1.835	0.7882
**5**	17.14	1.2341	0.1363	−0.0228	3.408	0.7929
**6**	27.14	1.4337	0.1084	−0.0186	0.4471	0.7426
**7**	52.86	1.7231	0.085	−0.0252	1.7969	0.7185
**8**	100.71	2.0031	0.0925	−0.0387	1.2213	0.785
**9**	47.14	1.6734	0.1133	−0.0336	4.4357	0.7926
**10**	35.71	1.5528	0.1022	−0.0346	5.6513	0.7723
**11**	48.57	1.6864	0.0192	−0.0089	0.1361	0.7008
**12**	18.57	1.2688	0.0576	−0.0097	0.2772	0.6591
**13**	12.86	1.1091	0.1368	−0.0135	0.6804	0.7483
**14**	12.86	1.1091	0.162	−0.019	1.0966	0.7271
**15**	21.43	1.331	0.0888	−0.0226	3.0572	0.7124
**16**	70	1.8451	0.1415	−0.0367	2.1767	0.752
**17**	17.14	1.2341	0.1391	−0.0266	2.3122	0.7225
**18**	50	1.699	0.0587	−0.0143	1.1376	0.7302
**19**	55.71	1.746	0.0575	−0.0274	0.4357	0.6287

**Note: ID**: compound number; **ESP**: electrostatic potential; **FNSA-3**: fractional atomic charge weighted partial negative surface area; **TMSA**: total molecular surface area; **PNSA-3:** total charge weighted partial negatively charged molecular surface area; SAMPOS*RPCG is the result of the partial surface area multiplied by the relative positive charge; * represents multiplier.

**Table 2:** Antifungal activity ratios (AR) and descriptors of cinnamaldehyde analogues for *Gloeophyllun trabeum*.

ID	AR	logAR	ESP-Min Net Atomic Charge for a H Atom, d1	ESP-RPCS Relative Positive Charged SA (SAMPOS*RPCG), d5	FNSA-3 (PNSA-3/TMSA), d6	FNSA-3 Fractional PNSA (PNSA-3/TMSA), d7
**1**	100	2	0.0585	1.3522	−0.0837	−0.0241
**2**	33.65	1.5073	0.0966	0.8218	−0.0635	−0.0201
**3**	43.78	1.6413	0.1701	0.7785	−0.0932	−0.0271
**4**	18.65	1.2706	0.1632	1.835	−0.0987	−0.0213
**5**	19.46	1.2891	0.1363	3.408	−0.0795	−0.0228
**6**	30.81	1.4887	0.1084	0.4471	−0.0629	−0.0186
**7**	55.41	1.7436	0.085	1.7969	−0.0773	−0.0252
**8**	68.65	1.8366	0.0925	1.2213	−0.0731	−0.0387
**9**	48.65	1.6874	0.1133	4.4357	−0.1226	−0.0336
**10**	35.68	1.5524	0.1022	5.6513	−0.1194	−0.0346
**11**	68.11	1.8332	0.0192	0.1361	−0.0354	−0.0089
**12**	43.38	1.6373	0.0576	0.2772	−0.0512	−0.0097
**13**	19.73	1.2951	0.1368	0.6804	−0.0625	−0.0135
**14**	15.41	1.1877	0.162	1.0966	−0.0726	−0.0190
**15**	29.73	1.4732	0.0888	3.0572	−0.0709	−0.0226
**16**	71.76	1.8559	0.1415	2.1767	−0.1262	−0.0367
**17**	23.24	1.3663	0.1391	2.3122	−0.0760	−0.0266
**18**	36.49	1.5621	0.0587	1.1376	−0.0424	−0.0143
**19**	51.89	1.7151	0.0575	0.4357	−0.0408	−0.0274

**Table 3:** Multilinear QSAR model obtained for cinnamaldehyde analogues for *Trametes versicolor* (*R*^2^ = 0.910, *F* = 35.32, and *s*^2^ = 0.0093).

Descriptor No.	*X*	Δ*X*	*t* Test Value	Name of Descriptor
0	−0.26082	0.4278	−0.6098	Intercept
1	−5.8562	0.6297	−9.3004	ESP-Min net atomic charge for a H atom, d1
2	−28.2750	3.3560	−8.4250	FNSA-3 Fractional PNSA (PNSA-3/TMSA), d2
3	−0.0912	0.0196	−4.6472	ESP-RPCS Relative charged SA (SAMPOS*RPCG) [Quantum-Chemical PC], d3
4	2.5481	0.6368	4.0017	YZ Shadow/YZ Rectangle, d4

**Table 4:** Multilinear QSAR model obtained for cinnamaldehyde analogues for *Gloeophyllun trabeum* (*R*^2^ = 0.926, *F* = 43.95, and *s*^2^ = 0.0049).

Descriptor No.	*X*	Δ*X*	*t* Test Value	Name of Descriptor
0	1.5166	0.0591	25.6824	Intercept
1	−5.8328	0.5080	−11.4819	ESP-Min net atomic charge for a H atom, d1
2	−0.1190	0.0170	−7.0087	ESP-RPCS Relative positive charged SA (SAMPOS*RPCG) [Quantum-Chemical PC], d5
3	−8.0388	1.3311	−6.0391	FNSA-3 (PNSA-3/TMSA) [Quantum-Chemical PC], d6
4	−11.201	2.8386	−3.9457	FNSA-3Fractional PNSA (PNSA-3/TMSA), d7

**Table 5:** Experimental logAR and Calculated logAR for *Trametes versicolor* and *Gloeophyllun trabeum*.

*Trametes versicolor*				*Gloeophyllun trabeum*			
ID	Exp.logAR	Calc.logAR	Difference	ID	Exp.logAR	Calc.logAR	Difference
**1**	2	1.9683	−0.0317	**1**	2	1.9568	−0.0432
**2**	1.4102	1.4807	0.0705	**2**	1.5073	1.5916	0.0843
**3**	1.4973	1.4345	−0.0628	**3**	1.6413	1.4842	−0.1571
**4**	1.2341	1.2272	−0.0069	**4**	1.2706	1.3783	0.1077
**5**	1.2341	1.2962	0.0621	**5**	1.2891	1.2106	−0.0785
**6**	1.4337	1.4827	0.049	**6**	1.4887	1.5455	0.0568
**7**	1.7231	1.6211	−0.1020	**7**	1.7436	1.7105	−0.0331
**8**	2.0031	2.1813	0.1782	**8**	1.8366	1.8532	0.0166
**9**	1.6734	1.641	−0.0324	**9**	1.6874	1.6901	0.0027
**10**	1.5528	1.5704	0.0176	**10**	1.5524	1.5953	0.0429
**11**	1.6864	1.6516	−0.0348	**11**	1.8332	1.7728	−0.0604
**12**	1.2688	1.3291	0.0603	**12**	1.6373	1.6672	0.0299
**13**	1.1091	1.1629	0.0538	**13**	1.2951	1.2905	−0.0046
**14**	1.1091	1.0807	−0.0284	**14**	1.1877	1.238	0.0503
**15**	1.331	1.394	0.063	**15**	1.4732	1.4575	−0.0157
**16**	1.8451	1.6645	−0.1806	**16**	1.8559	1.8572	0.0013
**17**	1.2341	1.3076	0.0735	**17**	1.3663	1.3394	−0.0269
**18**	1.699	1.557	−0.1420	**18**	1.5621	1.54	−0.0221
**19**	1.746	1.7395	−0.0065	**19**	1.7151	1.7642	0.0491

**Table 6:** Internal validation of the QSAR models of *Trametes versicolor* and *Gloeophyllun trabeum*.

Training Set	*N*	*R*^2^ (fit)	*F* (fit)	*s*^2^ (fit)	Test Set	*N*	*R*^2^ (pred)	*F* (pred)	*s*^2^ (pred)
Validation for the model in Table 3 *Trametes versicolor*									
A + B	13	0.909	20.07	0.0124	C	6	0.882	37.49	0.0350
A + C	12	0.946	30.72	0.0064	B	7	0.850	34.02	0.1102
B + C	13	0.930	26.55	0.0078	A	6	0.643	10.01	0.1474
Average		0.929	25.78	0.0089			0.792	27.17	0.0975
Validation for the model in Table 4 *Gloeophyllun trabeum*									
A + B	13	0.936	29.19	0.0057	C	6	0.766	17.32	0.0249
A + C	12	0.961	43.01	0.0035	B	7	0.812	26.93	0.0460
B + C	13	0.900	18.03	0.0056	A	6	0.920	58.79	0.0214
Average		0.932	30.08	0.0049			0.833	34.35	0.0308

**Table 7:** Antifungal activity ratios of designed compounds.

No		AR	Calc.logAR	Exp.logAR	Absolute Error
*Trametes versicolor*	A	100.92	2.0040	2.4767	0.4727
	B	101.86	2.0080	2.6561	0.6481
*Gloeophyllun trabeum*	A	153.70	2.1866	2.2022	0.0155
	B	161.74	2.2088	2.5390	0.3302
